# Non-linear Parameter Estimates from Non-stationary MEG Data

**DOI:** 10.3389/fnins.2016.00366

**Published:** 2016-08-22

**Authors:** Juan D. Martínez-Vargas, Jose D. López, Adam Baker, German Castellanos-Dominguez, Mark W. Woolrich, Gareth Barnes

**Affiliations:** ^1^Signal Processing and Recognition Group, Department of Electric and Electronic Engineering and Computation, Universidad Nacional de ColombiaManizales, Colombia; ^2^SISTEMIC, Facultad de Ingeniería, Universidad de Antioquia UDEAMedellín, Colombia; ^3^Oxford Centre for Human Brain Activity, Warneford Hospital, University of OxfordOxford, UK; ^4^Centre for Functional MRI of the Brain, John Radcliffe Hospital, University of OxfordOxford, UK; ^5^Wellcome Trust Centre for Neuroimaging, Institute of Neurology, University College LondonLondon, UK

**Keywords:** MEG inverse problem, co-registration, Hidden Markov Model, non-stationary brain activity, Bayesian comparison

## Abstract

We demonstrate a method to estimate key electrophysiological parameters from resting state data. In this paper, we focus on the estimation of head-position parameters. The recovery of these parameters is especially challenging as they are non-linearly related to the measured field. In order to do this we use an empirical Bayesian scheme to estimate the cortical current distribution due to a range of laterally shifted head-models. We compare different methods of approaching this problem from the division of M/EEG data into stationary sections and performing separate source inversions, to explaining all of the M/EEG data with a single inversion. We demonstrate this through estimation of head position in both simulated and empirical resting state MEG data collected using a head-cast.

## 1. Introduction

Typical MEG experimental design means that more data are recorded than analyzed. This is because M/EEG data is of such high dimension that we must restrict ourselves to experimental hypotheses, which focus on relatively narrow peri-stimulus time-frequency windows. Although the data outside of these windows may be of little use to address the experimental hypothesis, it still derives from the brain and can be useful.

Here we show how one might begin to harvest this kind of useful information from any MEG dataset. We combine two recent studies: Woolrich et al. (2013) who showed how that it is possible to break the MEG time series into a set of labeled stationary intervals; and Lopez et al. (2012) who showed it is possible to estimate the head position based on stationary MEG data. The estimation of head-position is attractive for three reasons—firstly it is non-linearly related to the measured data and therefore poses one of the most challenging estimation problems; secondly because using new recording techniques based on head-casts (Troebinger et al., 2014) we would be able to test our estimates against a ground truth; and finally because imprecise knowledge of head-location adds significant source of unmodeled error to the M/EEG source reconstruction problem (Lopez et al., 2012; Troebinger et al., 2014).

If the cortical location can be determined, other parameters that are also highly non-linear on the underlying current distribution can also be estimated. From any MEG dataset then in future, we can begin to harvest other information-like current density, cortical smoothness and so on-that can be used to update the prior information for estimating task-based data.

This paper proceeds as follows. We re-introduce the Hidden Markov Model (HMM) algorithm of Woolrich et al. (2013) to estimate stationary periods of MEG data. We then estimate the probability that these segments of MEG data derive from each point of the three-dimensional space of possible forward models (or head positions). We begin with a simulation study to demonstrate that the method works in principle. We then test the method using real data from a 10 min resting state recording in which the subject's head was fixed at a known position using a 3D printed head-cast (i.e., the empirical ground truth was known). We then compare the head position estimate based on our algorithms to this ground truth using either HMM segmentation, no-segmentation, or uninformed segmentation. We find that with all approaches we can estimate the location of the cortical mantle from MEG resting state data alone to within 2–3 mm or approximately half the cortical thickness.

## 2. Methods

**Figure 1:**
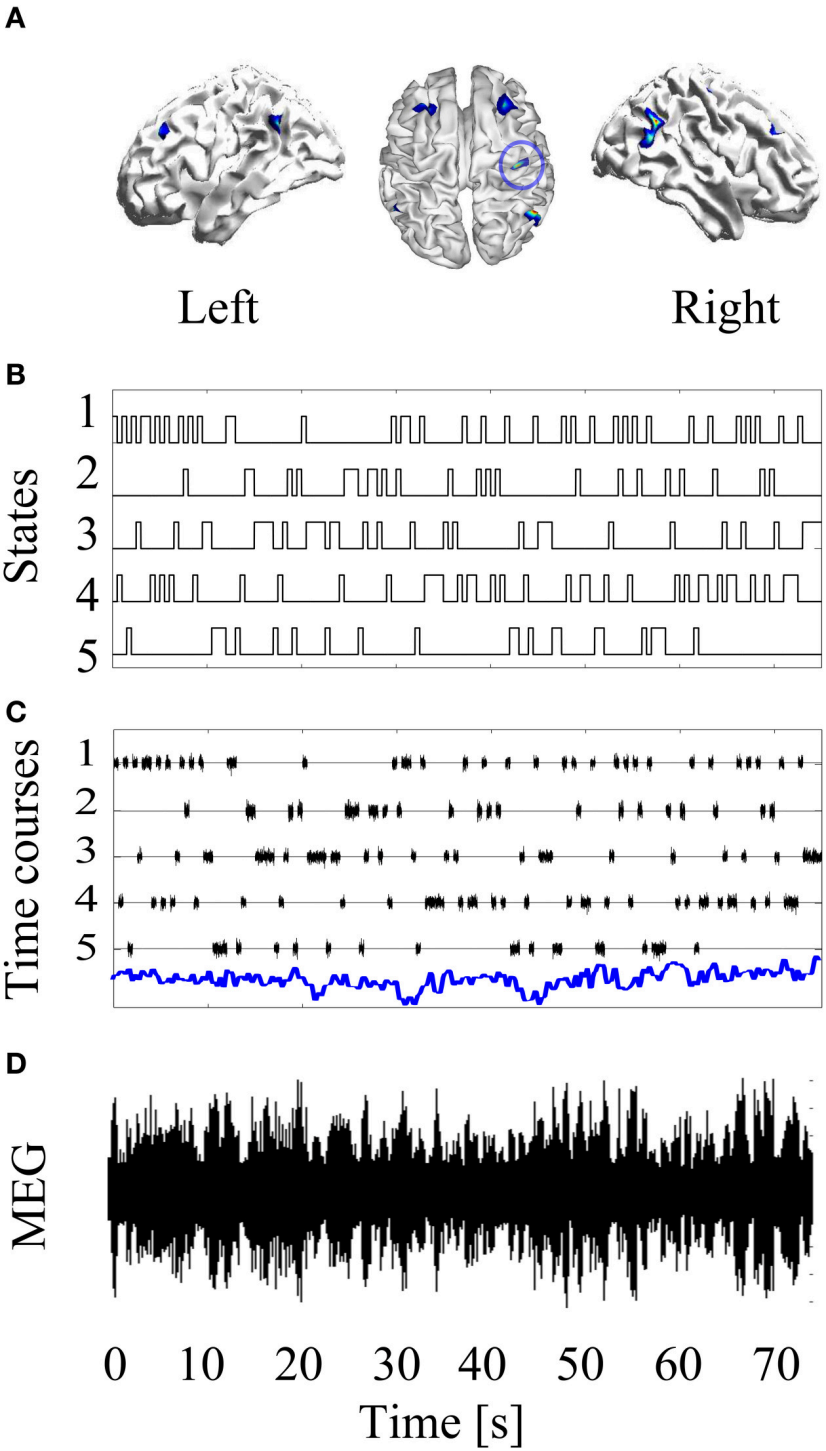
**Basic simulation set up: A stationary source of neural activity at the left primary motor cortex is simulated (blue circle in Panel A)**. Then, non-stationary confound sources were added to the forward modeling. The confound non-sources were activated and deactivated based on simulated HMM time courses **(B)**. Panel **(C)** shows the time courses of the neural activity (blue line) and the confound sources. After projecting this source configuration throughout the forward model, a non-stationary MEG recording is obtained **(D)**.

The basic simulation set up is laid out in Figure 1. There are 70 s of MEG data. The simulated source level data *J* comprise a single stationary source plus non-stationary contributions from five other sources. This gives rise to non-stationary sensor level data *Y*. The aim now is to blindly unpack this sensor level data in order to estimate the most-likely forward model (head-position) that generated it.

**Figure 2:**
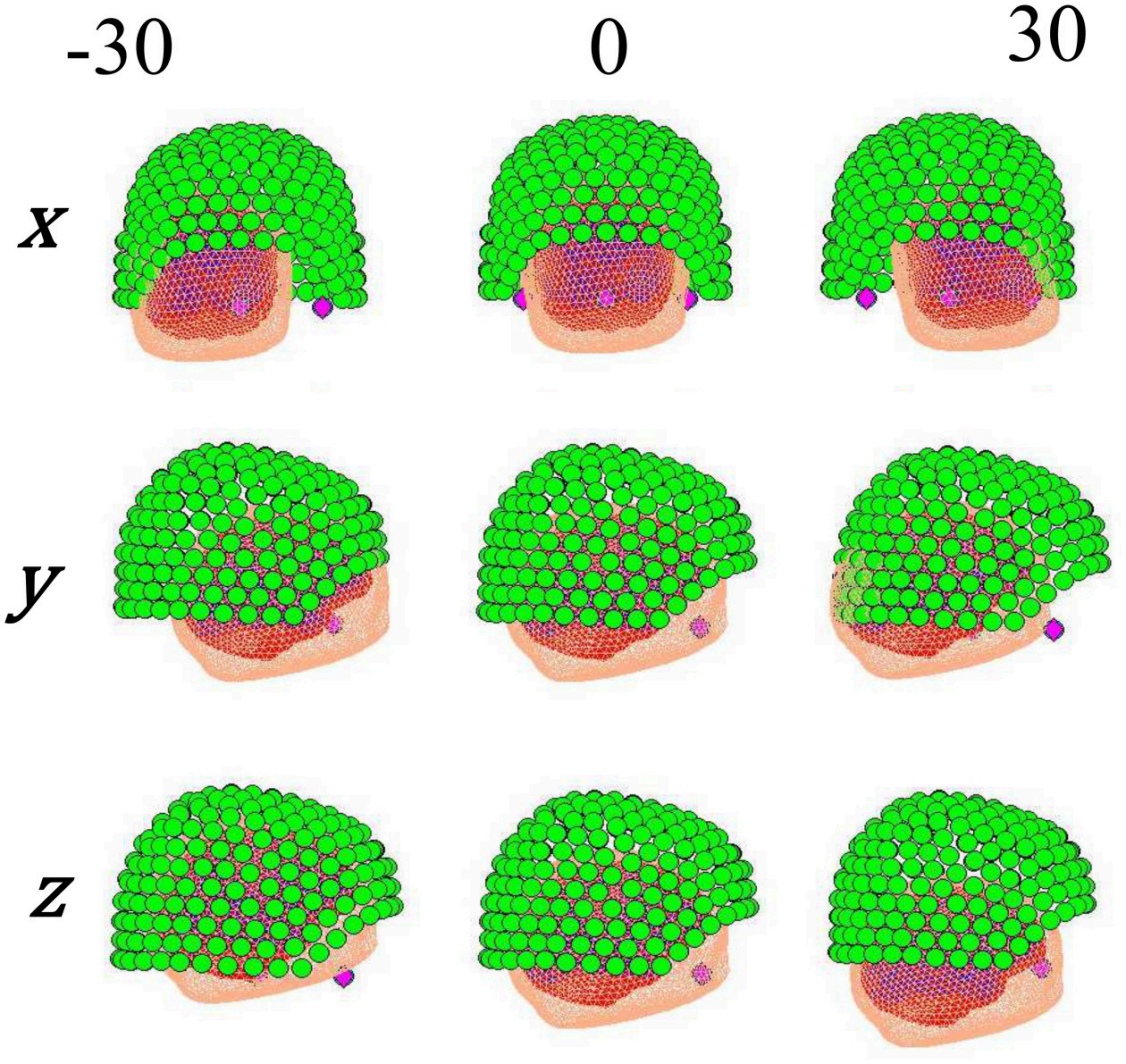
**Different locations given to the head position, controlled by moving the fiducials on each axis**.

Figure 2 shows the manipulation of the forward model. For some real or simulated MEG data we make an estimate of current flow on the cortical surface. We then displace the head with respect to the sensors and perform the same estimate again. Each current density estimate has an associated model evidence value (see below), and as the data remains the same (but the head position varies) we can directly compare model evidence values between source reconstructions at different head positions.

The estimation of head-position from stationary MEG data was outlined in a previous work (Lopez et al., 2012). The inversion scheme is a parametric empirical Bayes (PEB) routine implemented in the standard SPM framework. Empirical Bayes estimates the most likely priors based on the data. In the MEG case, these priors take the form of active cortical patches, which when combined provide an optimized source covariance matrix. One can make this estimate through a search across different possible patch combinations (here we optimized using a greedy search algorithm: GS, Friston et al., 2008). We then compare different ways of partitioning the data prior to the PEB stage. We do this in one of three ways: we assume stationarity and use all available MEG data to give a single inversion (and single model evidence value) per location; based on an HMM we classify the MEG data into *K* distinct self-similar stationary states and invert each of these data sections independently (providing *K* model evidence values per cortical location); or we use an uninformed segmentation to break the data into *K* segments (providing *K* model evidence values per cortical location).

In the remainder this section, we describe how the MEG data is partitioned into stationary segments using sensor level data, and we then go on to describe the source reconstruction assumptions and the model evidence metric we use to judge between inversions. Model evidence curves which peak at zero displacement will have higher accuracy, and the sharper the peak the higher the precision of this estimate.

### 2.1. Dynamic MEG segmentation based on hidden markov models (HMMs)

Let *Y* = {*y*_*t*_ : *t* ∈ *T*}, with *Y* ∈ ℝ^*C*×*T*^, the MEG data measured by *C* sensors at *T* time samples, where $y_{t}\in {\mathbb{R}}^{C\times 1}$ is the MEG sensor data at time instant *t*. Thus, we assume an HMM of length *T*, state space dimension *K* ∈ ℕ, and hidden state variables ***S*** = ***s***_1_, …, ***s***_*T*_, where the full true posterior probability of the model is given by:

$$P(S|Y)=P(s_{0}|\Pi_{0})\prod_{t}P(s_{t}|s_{t-1},\Psi )P(y_{t}|s_{t},\Theta )P(\Theta )P(\Pi ),$$

where *P*(**Θ**) and *P*(**Ψ**) are chosen to be non-informative priors, and *P*(***s***_0_|**Π**_0_) is the initial state probability. Furthermore, **Π** ∈ ℝ^*K*×*K*^ is the transition probability matrix *P*(***s***_*t*_|***s***_*t*−1_), where each element (*k,j*); ∀*j, k* = 1, …, *K* describes the probability of transition from states *k* to *j* within the time-intervals *t* − 1 and *t*, respectively. The term *P*(***y***_*t*_|***s***_*t*_, **Θ**) is the observation model that describes the distribution of the data for each of the states ***s***_*t*_. We assume that the observation model for state *k* is a multivariate Normal distribution **Θ**_*k*_ = {**μ**_*k*_, **Σ**_*k*_}, where **μ**_*k*_ ∈ ℝ^*K*×1^ is the mean vector, and $\sum_{k}\in {\mathbb{R}}^{K\times K}$ is the covariance matrix.

In this case we selected *K* for both simulated and real data to be the elbow of the singular values curve computed from the data covariance matrix ***YY***^⊤^.

We choose the most probable *a posteriori* state at each time point, *u*_*t*_ ∈ ℝ, using Viterbi decoding (Rezek and Roberts, 2005; Woolrich et al., 2013):

$$u_{t}=\underset{\forall k\in K}{\arg\max}\left\{P\left(s_{t}=k|Y\right)\right\}.$$

The resulting HMM state time courses can then be used to pool the data over distinct and potentially short-live periods on time to compute time-varying data covariance matrices, as follows:

$$C_{u\left(t\right)}=C_{\left(u\left(t\right)=k\right)}=\frac{1}{T_{k-1}}\overset{T_{k}}{\underset{j=1}{\sum }}\left(Y_{k}-{\overset{-}{Y}}_{k}\right){\left(Y_{k}-{\overset{-}{Y}}_{k}\right)}^{⊤}$$

where ***Y***_*k*_ comprises the time-instants for which the state *k* is the most probable, *T*_*k*_ is the length of ***Y***_*k*_, and ${\overset{-}{Y}}_{k}\in {\mathbb{R}}^{C\times 1}$ is the mean over those time points.

### 2.2. Bayesian MEG inverse problem

For the sake of simplicity, we will now drop notation depicting data from each MEG segment, and describe the inversion procedure for any segment of magnetic field data ***Y***. Where ***Y*** is given by (Grech et al., 2008; Dale and Sereno, 1993):

Y= LJ+Ξ,

where ***J*** ∈ ℝ^*D*×*T*^ is the amplitude of the *D* current dipoles distributed through the cortical surface with fixed orientation perpendicular to it, and ***L*** ∈ ℝ^*C*×*D*^ (commonly termed *lead field matrix*) is a gain matrix representing the relationship between sources and MEG data. We assume that the MEG measured data are affected by a zero mean Gaussian noise **Ξ** ∈ ℝ^*C*×*T*^ with covariance cov(**Ξ**) = *Q*_**Ξ**_ = λ_**Ξ**_***I***_*C*_, with $I_{C}\in {\mathbb{R}}^{C\times C}.$

Generally, source estimation can be expressed by the expected value of the posterior source activity distribution, which can be computed from the input data using the Bayes' theorem, as follows:

P(J|Y)=P(Y|J)P(J)/P(Y).

As outlined in Lopez et al. (2012), we can incorporate uncertainty about anatomical assumptions of the head model, that is, *L* = *L*(*h*), where *h* denotes the set of anatomical parameters (in this case the head-position data). Thus, the source reconstructed solution is rewritten in the form:

(1) $$\begin{array}{l} P\left(J|Y,h\right)=\frac{P\left(Y|J,h\right)P\left(J|h\right)}{P\left(Y|h\right)}, \end{array}$$

where the probability *P*(***Y***|*h*) (termed *evidence*) makes explicit the relationship between source reconstructed solutions and assumed anatomical parameters. Here, we consider *h* as the head location inside the MEG device. Therefore, provided the *n*-th head model, *h*_*n*_, implying ***L*** = ***L***(*h*_*n*_), we can solve Equation (1) by assuming that *J* is a zero mean Gaussian process with prior covariance cov(***J***) = ***Q***, with ***Q*** ∈ ℝ^*D*×*D*^. Thus, brain activity estimation, $\hat{J},$ is carried out by solving the widely known maximum-a-posteriori problem in the form:

(2) $$\begin{array}{ll} \hat{J}=\underset{J}{\arg\max}\left\{p\left(J|Y,h_{n}\right)\right\}=\underset{J}{\arg\max}\left\{p\left(Y|J,h_{n}\right)p\left(J,h_{n}\right)\right\}, & \; \end{array}$$

The optimization problem of Equation (2) yields the estimate $\hat{J}=\;{QL{\left(h\right)}_{n}}^{\text{T}}{\left(Q_{\Xi }+\;L{\left(h\right)}_{n}{QL{\left(h\right)}_{n}}^{\text{T}}\right)}^{-1}Y$ that requires prior knowledge about the sensor noise covariance ***Q***_**Ξ**_ and the source covariance matrix ***Q***. In order to supply the sensor noise covariance, we set ***Q***_**Ξ**_ = exp(λ_**Ξ**_)***I***_*C*_ where $I_{C}\in {\mathbb{R}}^{C\times C}$ is an identity matrix scaled by a hyper parameter modulating the sensor noise variance λ_**Ξ**_ (Phillips et al., 2002). The source covariance matrix is constructed as a sum of a set of *P* patches {***Q***_*p*_, *p* = 1, …, *P*} each one reflecting one potentially activated region of cortex weighted by the respective hyperparameter λ_*p*_, as follows (Friston et al., 2008; Belardinelli et al., 2012):

(3) $$\begin{array}{l} Q=\overset{P}{\underset{p=1}{\sum }}\exp\left(\lambda_{p}\right)Q_{p}. \end{array}$$

### 2.3. Assessment quality measure of source estimation solutions

To estimate the hyperparameter set determining the best covariance weight of a given head location *h*_*n*_, we use the so termed free energy:

(4) $$\begin{array}{l} F{\left(h\right)}_{n}=-\frac{T}{2}\mathrm{tr}(\Delta^{-1}C)-\frac{T}{2}\ln|\Delta |-\frac{CT}{2}\ln2\Pi \\ \;-\frac{1}{2}{\left(\mu -\eta \right)}^{⊤}\Omega^{-1}(\mu -\eta )+\frac{1}{2}\ln|ϒ\Omega^{-1}|, \end{array}$$

where **Δ** ∈ ℝ^*C*×*C*^ is the estimated model covariance, computed as $\Delta =\;{L{\left(h\right)}_{n}\;QL{\left(h\right)}_{n}}^{\text{T}}+\;Q_{\Xi }$; ***C*** ∈ ℝ^*C*×*C*^ is the measured data covariance, **μ** ∈ ℝ^*P*×1^ is the vector of prior means on the hyperparamters, **η** ∈ ℝ^*P*×1^ is the posterior means on the hyperparameters. **ϒ** ∈ ℝ^*P*×*P*^ is the posterior covariance of the hyperparameters, and **Γ** ∈ ℝ^*P*×*P*^ is the a prior covariance of hyperparameters {λ_*p*_}. Also, *T* and *C* are the number of temporal and spatial modes, respectively. Therefore, the Free Energy estimated in Equation (4) can be considered as the difference between the model accuracy (the first two terms) and the model complexity (the last two terms). The Free Energy can be maximized using standard variational schemes such as Expectation Maximization (EM) (Friston et al., 2008; Wipf et al., 2010).

In order to perform this optimization scheme we use a greedy search (GS) algorithm. The priors used to form the set of covariance components in the GS scheme were those implemented in the Statistical Parametric Mapping (SPM12) software package. That is, we used 512 covariance components with selected columns of a Greens function covering the entire cortical surface (see Lopez et al., 2014 for implementation details). Further, the set of GS hyperparameters were tuned through the Restricted Maximum Likelihood (ReML) algorithm, as explained and detail in Belardinelli et al. (2012) and Friston et al. (2008).

### 2.4. Fixed effect analysis

The problem is now to aggregate these model evidence values (*K* per-position for HMM and ST-GS, 1 per position for stationary case) across the different dimensions of the optimization (in this case *x*, *y*, and *z*). Here, we make use of the formalism behind Bayesian model comparison for families (Penny et al., 2010). This allows us to treat the multiple free energy values in the different dimensions as different model families. For these analyses we successively divided our parameter space in families of x, y, and z coordinates (steps of 5 mm) in each dimension; and therefore, they were able to produce posterior probability maps with one or two of the dimensions marginalized out.

## 3. Experiments

### 3.1. Simulated data description

Simulations were carried out using MEG system geometry based on the third order synthetic gradiometer configuration of a 274 (275 with one channel disabled) channel whole head CTF MEG system. The location of the brain anatomy with respect to the sensors was taken from a real experimental recording. For source space modeling, we made use of a tessellated surface of the gray-white matter interface with 8196 vertices (possible source localizations), with source orientations fixed and perpendicular to the surface. The leadfields were computed using a single-shell volume conductor (Nolte, 2003). Simulated data were generated with a sampling rate of 200 Hz and length of 75s. A dipolar source located in the left primary motor cortex at the MNI coordinate [41, −25, 49] mm was simulated. This source time course comprised Gaussian random noise with unit standard deviation [the units here are arbitrary (AU) as the SNR is specified by the simulated sensor level noise]. To generate non-stationary activity, five confound sources, located at the MNI coordinates in Table 1, were also simulated using a five state HMM with a transition probability of 0.25 between all states. Thus, when state *k* was active, the source time course corresponding to the *k*-th confound source was sampled from Gaussian random noise with standard deviation equal to 10 (AU), and otherwise the source time course had zero standard deviation. Additive Gaussian noise equivalent to the r.m.s. signal power was added in sensor space to give an effective Signal-to-Noise Ratio (SNR) of 0dB. Simulation scheme is shown in Figure 1.

**Table 1:** **MNI coordinates used for the confound sources in the simulated data**.

**State (#)**	**MNI coordinate (mm)**
1	50, −62, 26
2	−50, −62, 26
3	26, 32, 40
4	−26, 32, 40
5	−4, 50, 14

**Figure 3:**
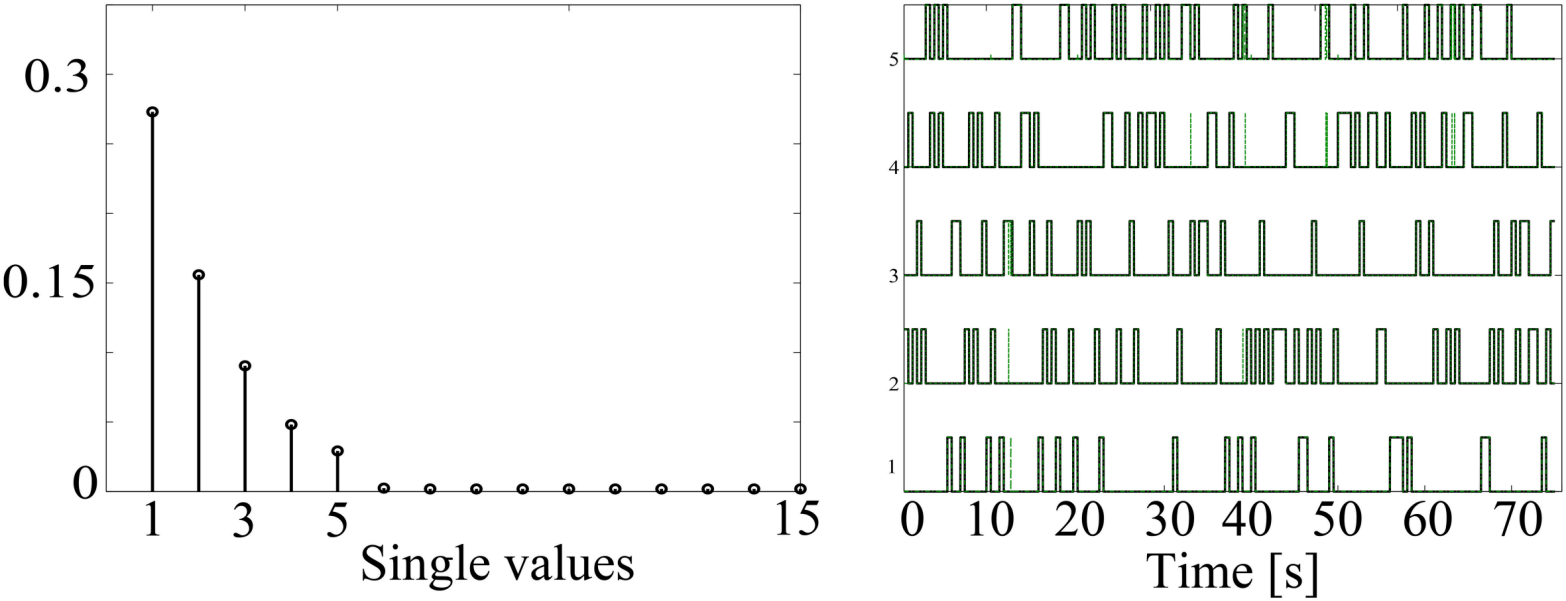
**Example of data simulated to contain time-varying sources using a 5 state HMM**. The singular values of the data covariance matrix that were used to estimate the number of states are shown in the **left** panel. Additionally, the simulated (black line) and estimated (dashed green line) HMM state time courses are overlapped in the **right** panel.

In order to illustrate the capability of the HMM to identify the underlying non-stationary activity of MEG data, Figure 3 shows a simulation with five confound sources. As explained above, the number of states was set according to the singular values of the data covariance matrix, which are shown in the left panel. As expected, the number of singular values with significant amount of energy corresponds to the same number of simulated confound sources. Also, the simulated (black line) and obtained (dashed green line) HMM time courses are shown overlapped in the right panel. There is a high correspondence between the simulated and reconstructed states, achieving correlation values around 0.95 for all the states.

### 3.2. Experiment description

To carry out a direct comparison of the stationary and non-stationary assumptions, we explored the influence of the head movement along a single axis (i.e., *x*, *y*, or *z*) taking as starting point the ground truth, which was accurately calculated with subject-specific head-casts produced using 3D printing (Troebinger et al., 2014). Afterwards, the head placement was varied within a ±30 mm interval centered at its original position with steps of 5 mm at time, as shown in Figure 2. In this way, the actual position of the head can be objectively compared against the position obtained with each method.

As the forward model is free to vary in this case, it is necessary to generate a new lead field matrix at each head location. Given the lead fields and the data covariance matrix (estimated under stationary or non-stationary assumptions), it is possible to make an estimate of *J* on the (displaced) cortical surface in addition to a model evidence value.

We consider three possible analyses which, depending on the stationarity assumptions, deliver either 1 or *K* free energy values per head position:

1. For the HMM-GS, we used the HMM to break up the *N* sample dataset into *K* distinct data segments. Each data segment comprised the *T*^*k*^ time points for which state *k* was the most probable. This gave *K* Free energy values per head position. For the stationary and the ST-GS cases (2 and 3 below), we selected the length (*p* samples) of successive time windows segments as *T*/*K* to again have *K* distinct data segments.
2. For the short-time GS (denoted as ST-GS), we did not use the HMM but broke the data up into *K* successive windows. For each of the *K* windows we made a source level estimate. This gave *K* free energy values per head position.
3. For the stationary case, we computed *K* covariance matrices obtained from each of the *K* windows from the stationary case above, and averaged them together in order to obtain a single covariance estimate. This gave a single Free energy value per dataset.

**Figure 4:**
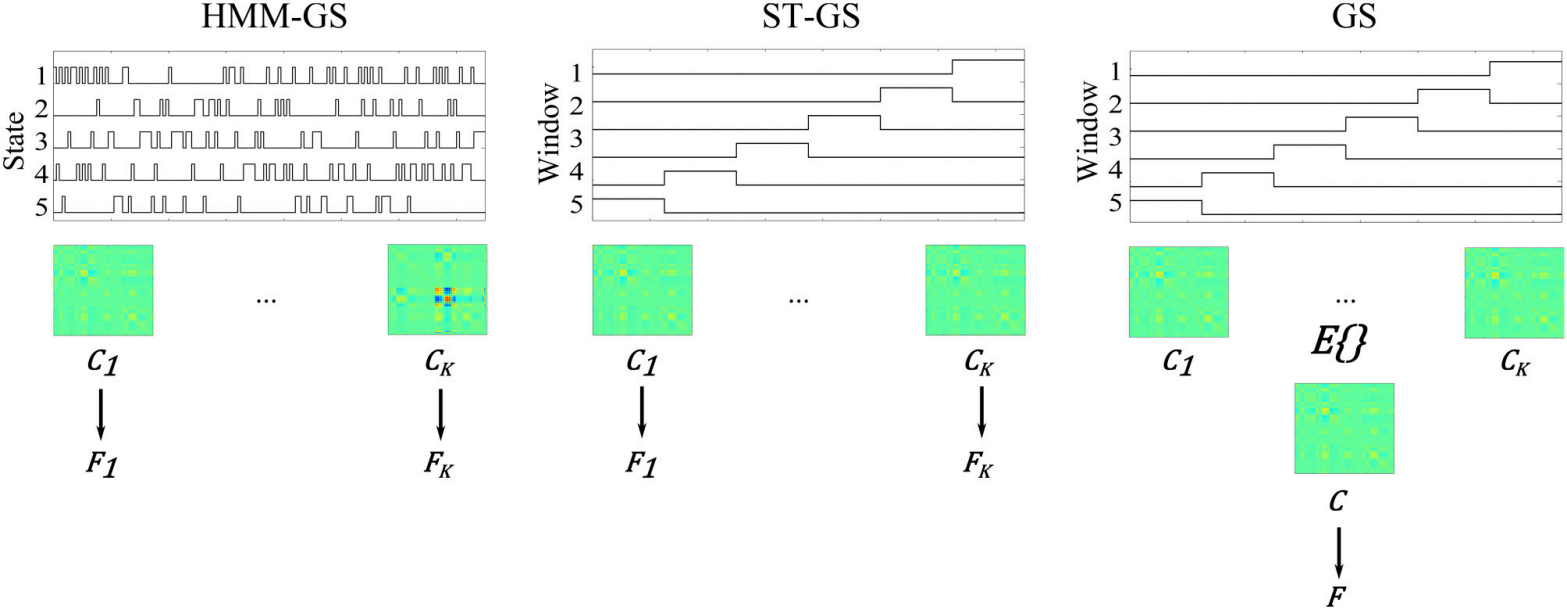
**Schematic representation of each stochastic assumption about MEG data**. The stochastic assumptions as explained above are schematized. **Left**: K estimated state time courses and their respective covariance matrices and free energy values; **Middle**: estimated short time windows, and their respective covariance matrices and free energy values; **Right**: Estimated covariance and free energy value for the stationary assumption.

These three possible schemes are outlined schematically in Figure 4.

### 3.3. Simulation results

**Figure 5:**
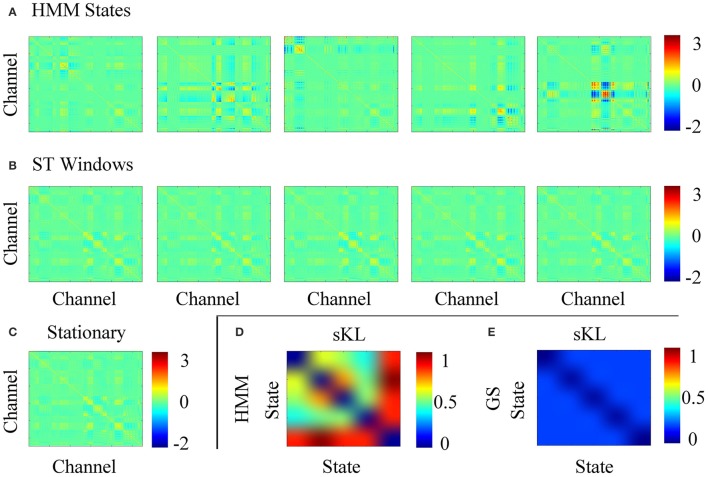
**(A)** Data covariance matrices computed by pooling the data during the points in time where those states are active. **(B)** Data covariance matrices computed in each stationary window. **(C)** Averaged data covariance matrix. **(D,E)** The symmetric KL computed between all five covariance windows for HMM and ST-GS, respectively.

Figure 5 shows the computed covariance matrices for each stochastic assumption, i.e., Figure 5A for each HMM state, Figure 5B for each short time window and Figure 5C for the full data. The data covariance matrices obtained for the HMM states are distinct from one another unlike the short time strategy that shows very similar covariance estimates for all windows. Additionally, to obtain an objective comparison between the covariance matrices, we used the **Symmetrised Kullback-Leibler (KL) divergence**, which provides a measure of dissimilarities for the different states *k, j* (Woolrich et al., 2013):

$$S_{KL}(k,j)=0.5\mathrm{tr}(\Sigma_{j}^{-1}\Sigma_{k})+0.5\mathrm{tr}(\Sigma_{k}^{-1}\Sigma_{j})-2C,$$

where larger values of *S*_*KL*_(*k, j*) indicate larger differences in the covariance matrices. Figures 5D,E show the symmetric KL computed between all five states and windows, respectively, where it can be seen that there are minimal differences between the covariances estimated with the short time strategy. However, the larger differences found between the covariance matrices estimated for the HMM suggest that the data were successfully decomposed into distinct dynamic components.

**Figure 6:**
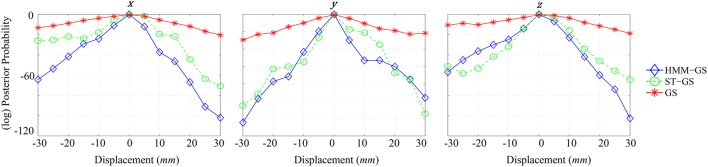
**Graphs of different normalized model evidence trajectories for a single axis with 5 mm of resolution, for simulated data with SNR = 0 dB**. **Left**: *x*-axis, **Middle**: *y*-axis, **Right**: *z*-axis.

Figure 6 shows (log) posterior probability computed for each direction by marginalizing over the complementary cardinal dimensions. As seen for *x*-axis (left), all curves put the most likely position of the head at the origin, although no prior knowledge of this location was used in the simulations. Comparing stationary with non-stationary assumptions, it is clear that the non-stationary (HMM and ST-GS) analysis gave rise to a steeper function or a higher precision estimate of the true underlying head-position. The *y* and *z* displacements show a similar picture. We were surprised that the arbitrarily segmented ST-GS algorithm performed almost as well as the data-driven segmentation (HMM-GS). We speculate that this could be due to a ceiling effect in which both algorithms perform well despite the imperfect data segmentation. We note however, when comparing the estimated covariance matrices that these estimates are based (Figure 5) that we might expect more robustness from the HMM algorithm which is based on a number of distinct states (rather than a single repeated state).

### 3.4. Resting state data

The empirical data were collected within a CTF 275 channel Omega system whilst the subject wore a nylon headcast machined to fit the subject's scalp, and provides an independent estimate of cortical anatomy with respect to the MEG sensors (Troebinger et al., 2014). For modeling of the source space, we used a tessellated surface of the gray–white matter interface with 21401 vertices (possible source localizations), with source orientations fixed and being orthogonal to the surface. Data were sampled at 600 Hz with 150 Hz hardware anti-aliasing filters. The resting state data were obtained from a 10 min closed eyes recording. Finally, MEG data were frequency filtered into the beta (13–30 Hz) and gamma (60–90 Hz) frequency bands.

### 3.5. Results resting state data

**Figure 7:**
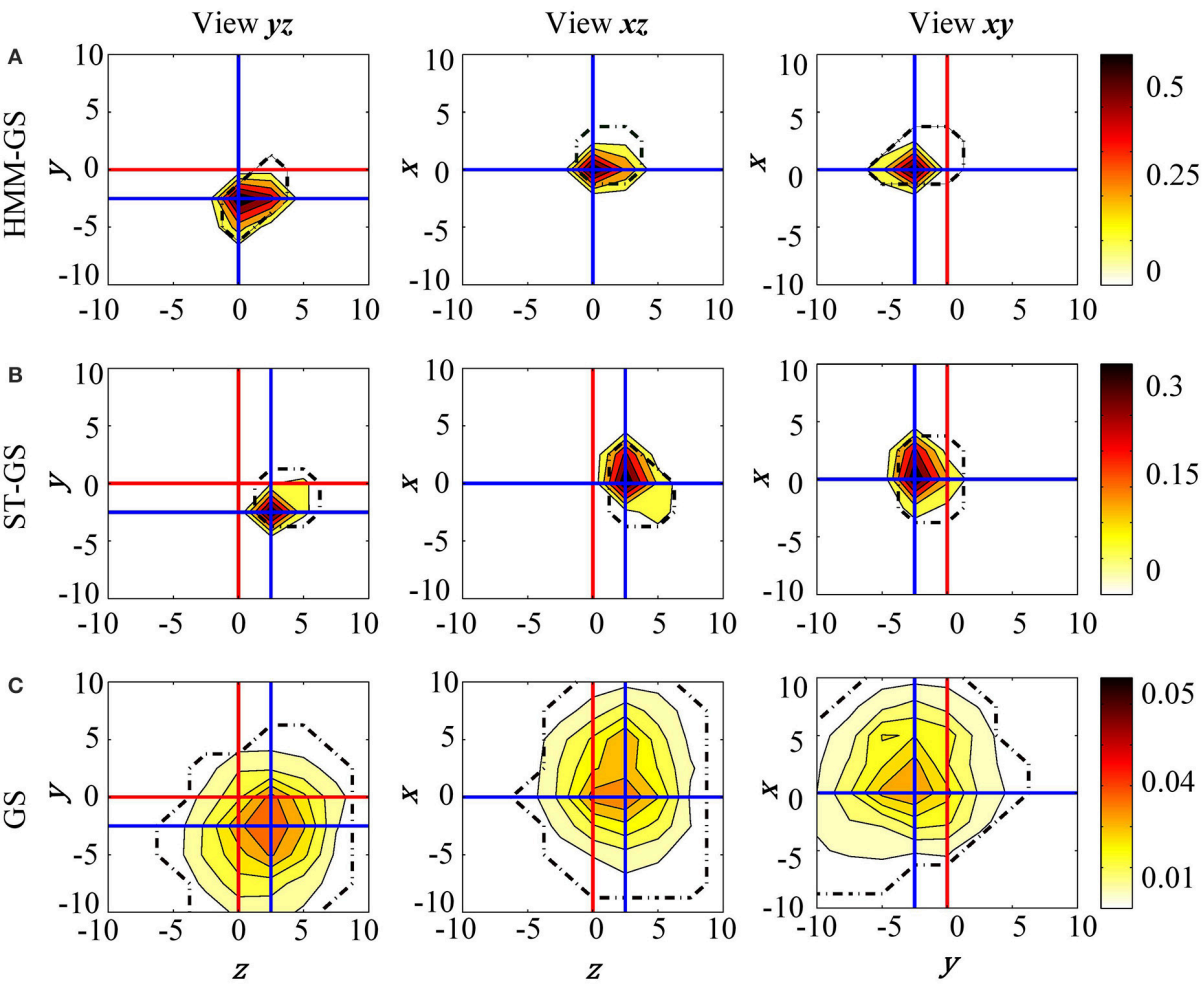
**Resting state raw data**. The posterior probability maps for the grid of head locations computed with **(A)** HMM-GS, **(B)** ST-GS, and **(C)** GS. HMM-GS from −10 to 10 mm with steps of 2.5 mm. The dashed black lines represent the 95% of the probability mass function.

The head model was moved in three possible directions (*x*, *y*, and *z* axes) from −10 to 10 mm in steps of 2.5 mm. Also, the number of states *K* was restricted to be the minimum value between the elbow of the singular values and 10, based on previous work using the HMM with resting state data (Woolrich et al., 2013). Figure 7 shows posterior probability maps estimated for the resting state data. The intersection of the red lines show the true (based on head-cast) estimated head position, while the intersection of the blue lines shows the peak of the posterior probability map (or the MEG data estimated head position) for three orthogonal views of the cube (*xy*, *xz*, and *yz*). Results show that with all three stationarity assumptions the MEG-estimated head positions were within around 2.5 mm of the head-cast estimated position. No algorithm estimated the head to be at (what we have assumed to be) the true location (0,0,0). We also looked at the same data filtered in 13–30 and 60–90 Hz bands; these results, which were less robust, are shown at a coarser (5 mm) scale in Figures S1–S3. In these cases of low SNR it would seem that the simplest approach GS (based on the average data covariance matrix) is the most robust. Additionally, we used a fixed proportion of the probability mass function (95 %) as probabilistic performance metric. With this measure we are able to objectively compare which of the methods provides a solution closer to the true location. The HMM-GS was the most accurate method, with the lowest distance to the true location, and also the proportion of the probability mass is closer to the origin coordinate, i.e., the probability distribution was more compact than the obtained with the ST-GS and GS methods.

## 4. Discussion

We have shown how it is possible to extract key source level parameters from resting state MEG data. We validated this approach empirically through estimation of head-position, where head-position constrained by a head-cast was known. The interesting thing about estimating head-position is that the problem is well-posed. A solution exists, it is stable to initial conditions, and the solution is directly verifiable from MEG recordings.

With all methods we were able to locate the cortical surface to within a 2.5–3.5 mm of where we believed the cortex to be based on our head-cast construction. Given that the cortical sheet which is between 2 and 5 mm thick and these estimates of head-position were made based purely on MEG data, we found this performance impressive. In order for the proposed methodology to work out, several assumptions have to be fulfilled: current flow must be normal to the cortical surface; the forward models must accurately describe the propagation of magnetic field; the inversion assumptions (in this case MSP) must be reasonable, and finally the goodness of fit metric (in this case Free energy) must be useful. In other words, as we improve upon functional and anatomical modeling assumptions, our estimate of head-position should become more accurate and precise. The formalism here is attractive as its performance is quantified in millimeters.

There are a number of possible sources of the residual displacement error. Notably the inversion scheme and the head-cast. We have considered only a single optimization scheme with a single set of cortical patches (or priors); it may be that these patches are not physiologically reasonable (see below) or are sub-optimally located and hence bias the location estimate. Here we estimated the three head-location parameters for simplicity. In previous work (Lopez et al., 2012) we estimated both head position and orientation. We see no reason why all six parameters could not be estimated in practice. Indeed it is possible that some of the deviation between our ground-truth and our estimate of head-position is due to imprecise head orientation. The head-cast also has errors associated with it—for example absolute errors in coregistration—due to the cortical surface extraction based on an MRI scan from a supine subject, whilst the MEG was performed seated.

In simulation and in the empirical tests we found relatively small differences between the informed (HMM-GS) and un-informed (ST-GS) data partitioning. This could be because despite imperfect partitioning the GS optimization scheme was able to identify the multiple temporally overlapping sources. For the empirical data we had also anticipated improved performance when looking at sub-bands as we thought the stationary states within a narrower frequency band would be simpler and hence easier to optimize. Because of this we used the HMM to identify stationary states in the same dataset but pre-filtered to the Beta (15–30 Hz) and Gamma (60–90 Hz) bands. To our surprise, we found the most robust estimation of head-position when we used the broad-band data. It is possible that for low SNR data any imperfect partitioning at the HMM stage simply adds noise to the signal.

It may seem that using complex Bayesian methods to demonstrate something as straightforward as head-position is excessive. We should point out that our main aim here was to estimate how non-linear parameter estimates could be made from non-stationary MEG data. We see the main utility of this approach as allowing us to answer questions about the underlying cortical structure that generated the MEG data. For example—with this approach we can mine large amounts of resting state data and ask if the MEG data in a specific frequency band more likely to have arisen from the superficial or deep cortical manifold (Bastos et al., 2012). One could imagine an algorithm that mines MEG records, first refining head position and then estimating the effect of different volume conductor models, different cortical patch extents, or even estimating cortical structure (Lopez et al., 2012).

It is now becoming clear that resting state brain activity can be thought of as an ongoing rehearsal of task related dynamics. Recent work by Brookes et al. (2014) for example showed how states identified at rest can explain much of the task related variance (O'Neil et al., 2014). If this resting state activity is a continuous and evolving rehearsal of useful task related brain states, we can make use of all these data to update our estimates of key electrophysiological parameters. Resting state activity is attractive as it is easy to measure consistently across sites and over long periods. This is especially exciting with the prospect of wearable MEG systems in the near future, where one might expect to have extremely long data recordings.

Another interesting avenue would be to use this method to test generic generative models of resting (and hence task) state dynamics. For example, algorithms like MSP can be used to compare specific source covariance priors corresponding to canonical resting state modes. This would provide a direct and compact parameterization that might explain away a considerable amount of MEG signal variance—as well as giving a better understanding of these networks in health and disease.

## Author contributions

JM did the experiments and helped to write the paper. JL helped to design the experiments and to interpret the results. AB supported the HMM implementation. MW helped to interpret the HMM-based results and to design the experiments. GC helped to write the paper. GB helped to design the experiments and to interpret all the obtained results.

### Conflict of interest statement

The authors declare that the research was conducted in the absence of any commercial or financial relationships that could be construed as a potential conflict of interest. The reviewer ZZ and handling Editor declared their shared affiliation, and the handling Editor states that the process nevertheless met the standards of a fair and objective review.

## References

[B1] Bastos A. M. Usrey W. M. Adams R. A. Mangun G. R. Fries P. Friston K. J. (2012). Canonical microcircuits for predictive coding. Neuron 76, 695–711. 10.1016/j.neuron.2012.10.038 23177956 PMC3777738

[B2] Belardinelli P. Ortiz E. Barnes G. Noppeney U. Preissl H. (2012). Source reconstruction accuracy of MEG and EEG bayesian inversion approaches. PLoS ONE 7:e51985. 10.1371/journal.pone.0051985 23284840 PMC3527408

[B3] Brookes M. J. O'Neill G. C. Hall E. L. Woolrich M. W. Baker A. Corner S. P.. (2014). Measuring temporal, spectral and spatial changes in electrophysiological brain network connectivity. Neuroimage 91, 282–299. 10.1016/j.neuroimage.2013.12.066 24418505

[B4] Dale A. M. Sereno M. I. (1993). Improved localization of cortical activity by combining EEG and MEG with MRI cortical surface reconstruction: a linear approach. J. Cogn. Neurosci. 5, 162–176. 10.1162/jocn.1993.5.2.162 23972151

[B5] Friston K. Harrison L. Daunizeau J. Kiebel S. Phillips C. Trujillo-Barreto N.. (2008). Multiple sparse priors for the M/EEG inverse problem. Neuroimage 39, 1104–1120. 10.1016/j.neuroimage.2007.09.048 17997111

[B6] Grech R. Cassar T. Muscat J. Camilleri K. P. Fabri S. G. Zervakis M.. (2008). Review on solving the inverse problem in EEG source analysis. J. Neuroeng. Rehabil. 5, 792–800. 10.1186/1743-0003-5-25 18990257 PMC2605581

[B7] López J. D. Litvak V. Espinosa J. J. Friston K. Barnes G. R. (2014). Algorithmic procedures for bayesian MEG/EEG source reconstruction in SPM. Neuroimage 84, 476–487. 10.1016/j.neuroimage.2013.09.002 24041874 PMC3913905

[B8] López J. D. Penny W. D. Espinosa J. J. Barnes G. R. (2012). A general bayesian treatment for MEG source reconstruction incorporating lead field uncertainty. Neuroimage 60, 1194–1204. 10.1016/j.neuroimage.2012.01.077 22289800 PMC3334829

[B9] Nolte G. (2003). The magnetic lead field theorem in the quasi-static approximation and its use for magnetoencephalography forward calculation in realistic volume conductors. Phys. Med. Biol. 48:3637. 10.1088/0031-9155/48/22/002 14680264

[B10] O'Neil E. B. Hutchison R. M. McLean D. A. Köhler S. (2014). Resting-state fMRI reveals functional connectivity between face-selective perirhinal cortex and the fusiform face area related to face inversion. Neuroimage 92, 349–355. 10.1016/j.neuroimage.2014.02.005 24531049

[B11] Penny W. D. Stephan K. E. Daunizeau J. Rosa M. J. Friston K. J. Schofield T. M.. (2010). Comparing families of dynamic causal models. PLoS Comput. Biol. 6:e1000709. 10.1371/journal.pcbi.1000709 20300649 PMC2837394

[B12] Phillips C. Rugg M. D. Friston K. J. (2002). Systematic regularization of linear inverse solutions of the EEG source localization problem. Neuroimage 17, 287–301. 10.1006/nimg.2002.1175 12482084

[B13] Rezek I. Roberts S. (2005). Ensemble hidden markov models with extended observation densities for biosignal analysis, in Probabilistic Modeling in Bioinformatics and Medical Informatics, eds Husmeier D. Dybowski R. Roberts S. Advanced Information and Knowledge Processing (London: Springer), 419–450.

[B14] Troebinger L. López J. D. Lutti A. Bradbury D. Bestmann S. Barnes G. (2014). High precision anatomy for MEG. Neuroimage 86, 583–591. 10.1016/j.neuroimage.2013.07.065 23911673 PMC3898940

[B15] Wipf D. P. Owen J. P. Attias H. T. Sekihara K. Nagarajan S. S. (2010). Robust Bayesian estimation of the location, orientation, and time course of multiple correlated neural sources using MEG. Neuroimage 49, 641–655. 10.1016/j.neuroimage.2009.06.083 19596072 PMC4083006

[B16] Woolrich M. W. Baker A. Luckhoo H. Mohseni H. Barnes G. Brookes M.. (2013). Dynamic state allocation for MEG source reconstruction. Neuroimage 77, 77–92. 10.1016/j.neuroimage.2013.03.036 23545283 PMC3898887

