# Supplementary material for: Non-linear Parameter Estimates from Non-stationary MEG Data
Source: Front Neurosci. 2016 Aug 22;10:366. doi: 10.3389/fnins.2016.00366 (PMC4993126; doi:10.3389/fnins.2016.00366)
Supplement: Supplementary file 1 [file Presentation1.pdf]

# *Supplementary Material*: Non-linear parameter estimates from non-stationary MEG data

**Juan David Martínez-Vargas[1*],Jose David López[2], Adam Baker[3], German Castellanos-Dominguez[1], Mark Woolrich[3,4] and Gareth Barnes [5]**
*Correspondence:
Juan David Martinez-Vargas
jmartinezv@unal.edu.co

## 1 SUPPLEMENTARY FIGURES

The following figures correspond to the same resting state data experiment carried out over a coarser mesh. Also, the resting state data was filtered into the Beta (13-30)Hz and Gamma (60-90)Hz frequency bands.

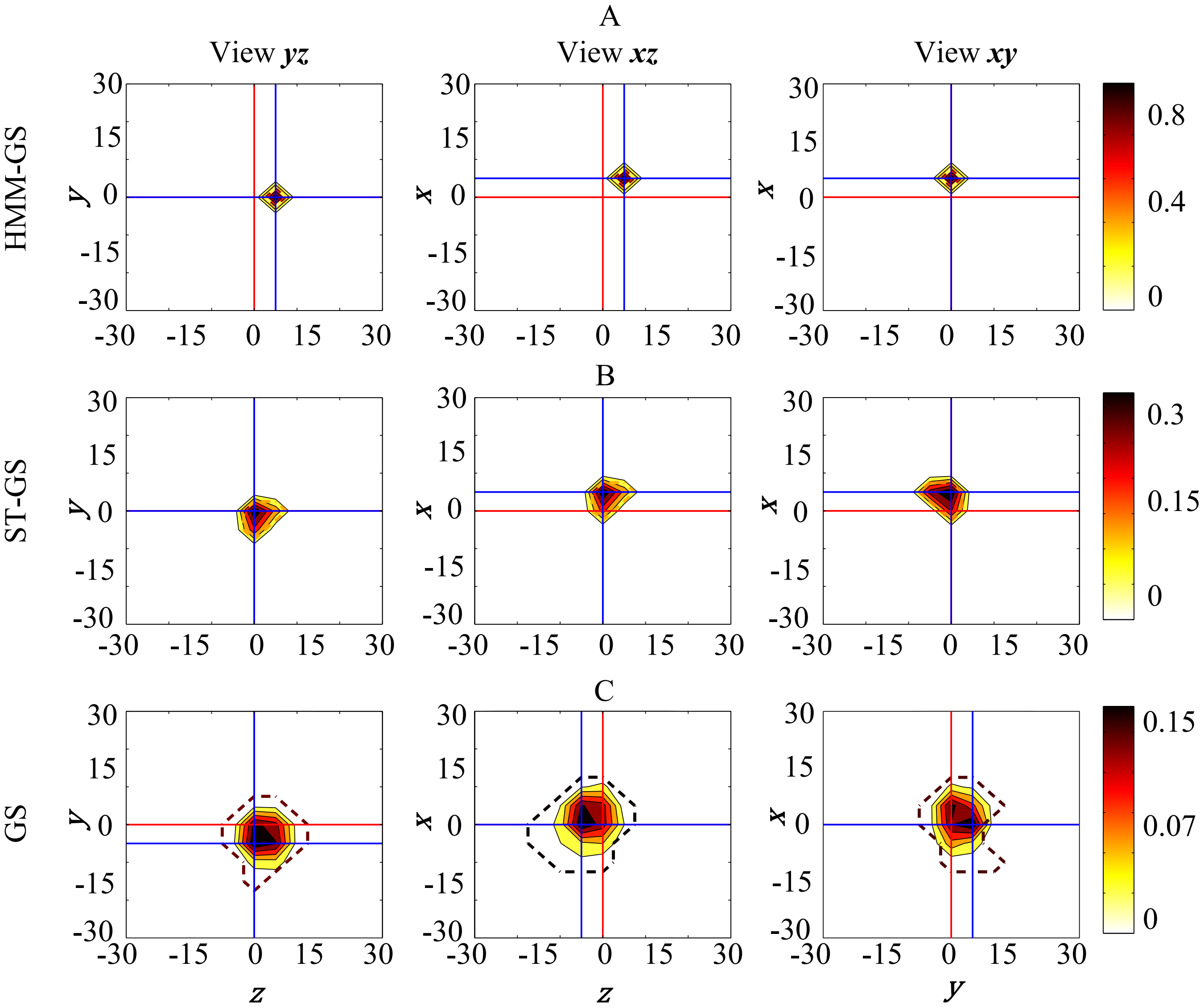


**Figure 1.** Resting state raw data. The posterior probability maps for the grid of head locations computed with (A) HMM-GS, (B) ST-GS, and (C) GS. HMM-GS from -30 mm to 30 mm with steps of 5 mm. The dashed black lines represent the 95 % of the probability mass function.

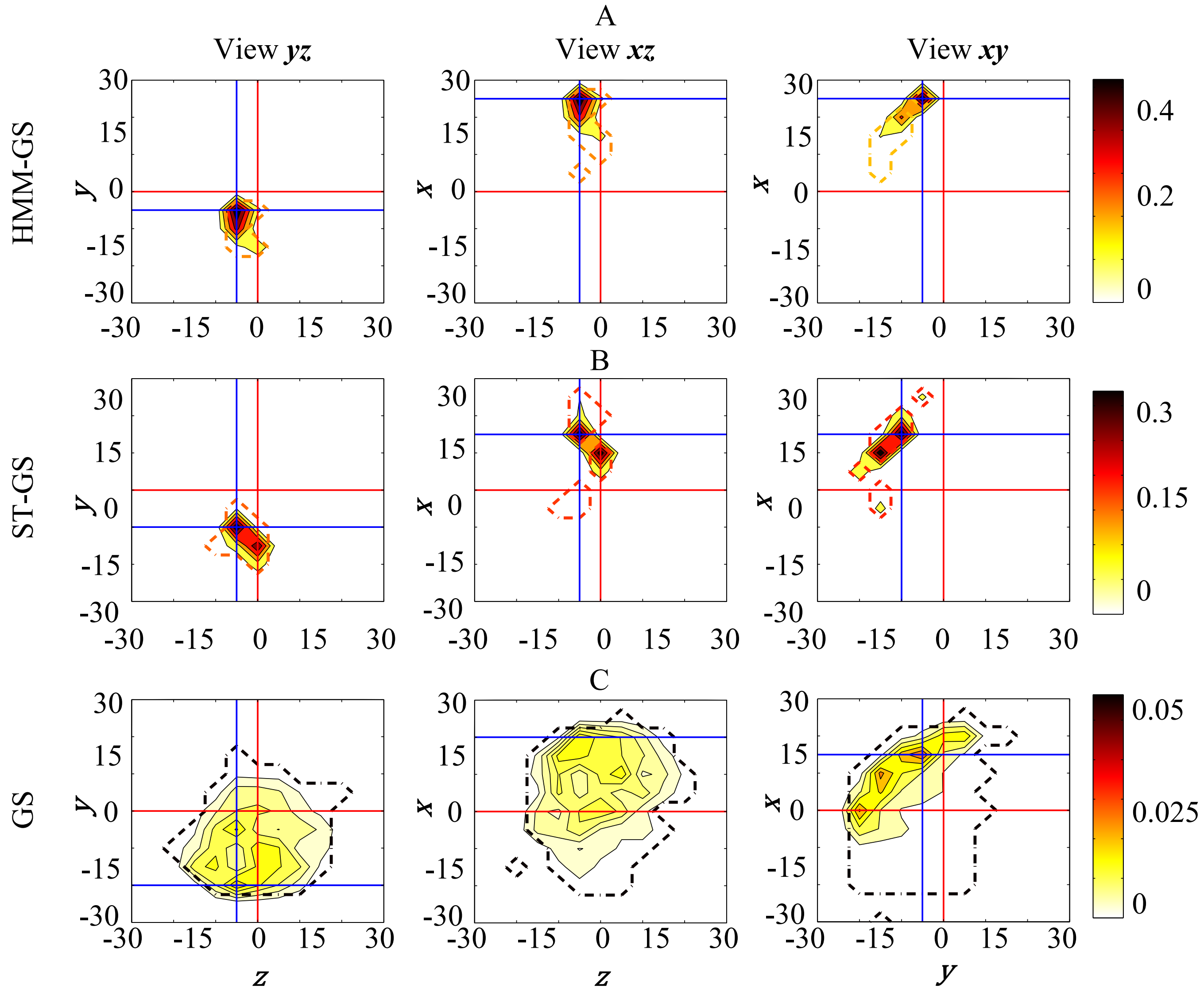


**Figure 2.** Resting state data filtered into 13-30 Hz ($\beta$ band). The posterior probability maps for the grid of head locations computed with (A) HMM-GS, (B) ST-GS, and (C) GS. HMM-GS from -10 mm to 10 mm with steps of 2.5 mm. The dashed black lines represent the 95 % of the probability mass function.

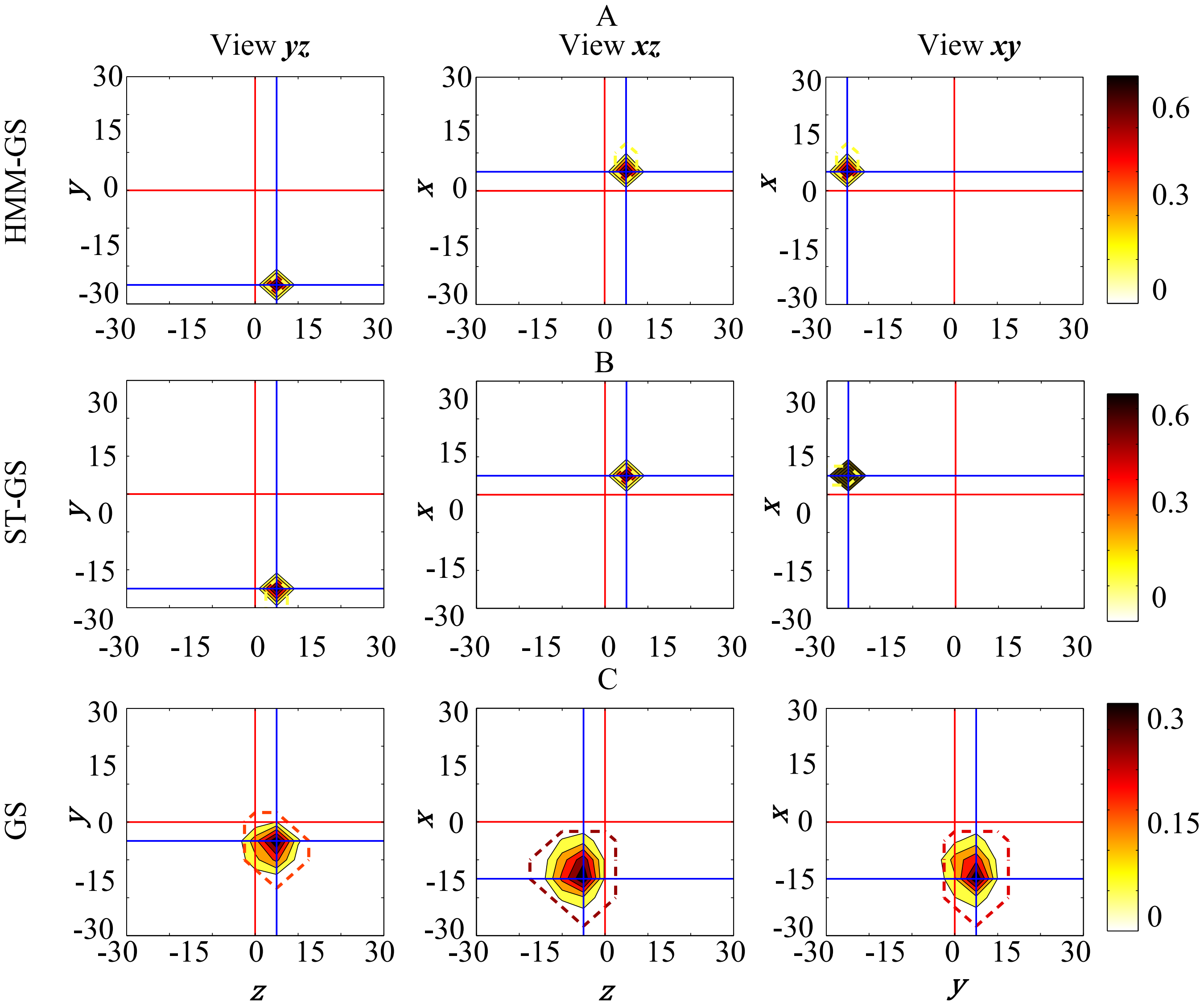


**Figure 3.** Resting state data filtered into 60-90 Hz ($\gamma$ band) . The posterior probability maps for the grid of head locations computed with (A) HMM-GS, (B) ST-GS, and (C) GS. HMM-GS from -30 mm to 30 mm with steps of 5 mm. The dashed black lines represent the 95 % of the probability mass function.
